# Psychotropic treatment in patients with arrhythmia: About a case

**DOI:** 10.1192/j.eurpsy.2021.1822

**Published:** 2021-08-13

**Authors:** M. Suárez-Gómez, S.S. Sánchez Rus

**Affiliations:** 1 Psychiatry, Unidade Local de Saúde do Baixo Alentejo, Beja, Portugal; 2 Jaén, Complejo Hospitalario Jaén, Jaén, Spain

**Keywords:** Depression, Psychotropic drugs, mental health and cardiovascular disease, arrythmia

## Abstract

**Introduction:**

It is knowed the relationship between psychological problems and cardiovascular disease. Pychological alterations can cause cardiovascular diseases, and a cardiovascular event can trigger psychological alterations.

**Objectives:**

The aim was to present a clinical case about a young man with cardiovascular alterations and depressive symptoms and to analyze the role played by cardiovascular drugs, psychoactive drugs, and their interactions.

**Methods:**

We present the clinical case and search the relation between cardiovascular disease and depressive symptoms and treatment at scientific literature of last five years.

**Results:**

A 38-year-old man comes to the emergency room with symptoms of palpitations, fatigue and shortness of breath for 2 weeks. An electrocardiogram is performed showing premature ventricular beats. The patient reports that he is sadder recently due to the loss of work, for which he is prescribed sertraline 50 mg daily and is referred to cardiology. No medical history or consumption of alcohol, tobacco or other toxins. The cardiologist requests ergometry, echocardiography, and Holter monitoring, resulting in all normal tests, with no evidence of ischemia. Bisoprolol 2.5 mg daily is prescribed and sertraline 50 mg daily is maintained. After two months, the patient reports feeling better in spirit. The control electrocardiogram is normal and the patient reports disappearance of palpitations. You are referred to your family doctor.

**Conclusions:**

Elevation of cortisol, platelet hyperactivity, and alteration in heart rate variability were found in depressives. The SSRIs would be the ones of choice. Dual serotonin and noraderaline reuptake inhibitors should be avoided. Other atypical drugs such as bupropion or trazodone should be considered.

**Disclosure:**

No significant relationships.

